# Opportunities for Pharmacogenetic Testing to Guide Dosing of Medications in Youths With Medicaid

**DOI:** 10.1001/jamanetworkopen.2023.55707

**Published:** 2024-02-13

**Authors:** Sonya Tang Girdwood, Matthew Hall, James W. Antoon, Kathryn E. Kyler, Derek J. Williams, Samir S. Shah, Lucas E. Orth, Jennifer Goldman, James A. Feinstein, Laura B. Ramsey

**Affiliations:** 1 Division of Hospital Medicine, Cincinnati Children’s Hospital Medical Center, Cincinnati, Ohio; 2 Division of Translational and Clinical Pharmacology, Cincinnati Children’s Hospital Medical Center, Cincinnati, Ohio; 3 Department of Pediatrics, University of Cincinnati College of Medicine, Cincinnati, Ohio; 4 Children’s Hospital Association, Lenexa, Kansas; 5 Department of Pediatrics, Vanderbilt University School of Medicine, Nashville, Tennessee; 6 Division of Hospital Medicine, Monroe Carell Jr Children's Hospital at Vanderbilt University Medical Center, Nashville, Tennessee; 7 Division of Hospital Medicine, Children’s Mercy Kansas City, Kansas City, Missouri; 8 Division of Clinical Pharmacology, Children’s Mercy Kansas City, Kansas City, Missouri; 9 School of Medicine, University of Missouri-Kansas City; 10 Division of Infectious Diseases, Cincinnati Children’s Hospital Medical Center, Cincinnati, Ohio; 11 Department of Clinical Pharmacy, University of Colorado Skaggs School of Pharmacy, Aurora; 12 Division of Infectious Diseases, Children’s Mercy Kansas City, Kansas City, Missouri; 13 Adult and Child Consortium for Health Outcomes Research and Delivery Science, Children’s Hospital Colorado, University of Colorado, Aurora

## Abstract

**Importance:**

There are an increasing number of medications with a high level of evidence for pharmacogenetic-guided dosing (PGx drugs). Knowledge of the prevalence of dispensings of PGx drugs and their associated genes may allow hospitals and clinical laboratories to determine which pharmacogenetic tests to implement.

**Objectives:**

To investigate the prevalence of outpatient dispensings of PGx drugs among Medicaid-insured youths, determine genes most frequently associated with PGx drug dispenses, and describe characteristics of youths who were dispensed at least 1 PGx drug.

**Design, Setting, and Participants:**

This serial cross-sectional study includes data from 2011 to 2019 among youths aged 0 to 17 years in the Marketscan Medicaid database. Data were analyzed from August to December 2022.

**Main Outcomes and Measures:**

PGx drugs were defined as any medication with level A evidence as determined by the Clinical Pharmacogenetics Implementation Consortium (CPIC). The number of unique youths dispensed each PGx drug in each year was determined. PGx drugs were grouped by their associated genes for which there was CPIC level A evidence to guide dosing, and a dispensing rate (No. of PGx drugs/100 000 youths) was determined for each group for the year 2019. Demographics were compared between youths dispensed at least 1 PGx drug and those not dispensed any PGx drugs.

**Results:**

The number of Medicaid-insured youths queried ranged by year from 2 078 683 youths in 2011 to 4 641 494 youths in 2017, including 4 126 349 youths (median [IQR] age, 9 [5-13] years; 2 129 926 males [51.6%]) in 2019. The proportion of Medicaid-insured youths dispensed PGx drugs increased from 289 709 youths (13.9%; 95% CI, 13.8%-14.0%) in 2011 to 740 072 youths (17.9%; 95% CI, 17.9%-18.0%) in 2019. Genes associated with the most frequently dispensed medications were *CYP2C9, CYP2D6*, and *CYP2C19* (9197.0 drugs [95% CI, 9167.7-9226.3 drugs], 8731.5 drugs [95% CI, 8702.5-8759.5 drugs], and 3426.8 drugs [95% CI, 3408.1-3443.9 drugs] per 100 000 youths, respectively). There was a higher percentage of youths with at least 1 chronic medical condition among youths dispensed at least 1 PGx drug (510 445 youths [69.0%; 95% CI, 68.8%-69.1%]) than among 3 386 277 youths dispensed no PGx drug (1 381 544 youths [40.8%; 95% CI, 40.7%-40.9%) (*P* < .001) in 2019.

**Conclusions and Relevance:**

In this study, there was an increasing prevalence of dispensings for PGx drugs. This finding suggests that pharmacogenetic testing of specific drug-gene pairs should be considered for frequently prescribed PGx drugs and their implicated genes.

## Key Points

**Question:**

What is the prevalence of dispensings for medications with a high level of evidence for pharmacogenetic-guided dosing (PGx drugs) among Medicaid-insured youths, and what are the most frequently implicated genes?

**Findings:**

In this cross-sectional study of more than 4.1 million youths receiving Medicaid in 2019, the percentage of included youths dispensed PGx drugs increased from 13.9% in 2011 to 17.9% in 2019. Genes associated with the most frequently dispensed medications were *CYP2C9, CYP2D6*, and *CYP2C19*.

**Meaning:**

This study found that dispensings for PGx drugs increased among Medicaid-insured youths, suggesting that targeted pharmacogenetic testing of specific drug-gene pairs should be considered to optimize prescribing.

## Teaser

This cross-sectional study investigates rates of dispensing of medications with a high level of evidence for pharmacogenetic-guided testing from 2011 to 2019 among youths with Medicaid.

## Introduction

Pharmacogenetics takes into account the influence of an individual’s genetic makeup on drug efficacy and toxic effects, allowing for personalized drug selection and dosing regimens with the goal of improving efficacy and reducing adverse events at an individual level.^1^ There are an increasing number of drugs with a high level of evidence for pharmacogenetic testing to guide prescribing, as indicated by the rapidly increasing number of guidelines published by the Clinical Pharmacogenetics Implementation Consortium (CPIC).^2^ This international consortium systematically grades evidence and clinical recommendations and publishes peer-reviewed gene-drug clinical practice guidelines. Since its inception in 2009, CPIC has published 26 guidelines, which provide recommendations for drug or dose changes based on genetic test results for 93 drugs with the highest level of evidence (level A).

Several institutions have implemented pharmacogenetic testing in their pediatric populations. In a survey of 13 pediatric institutions within the Children’s Hospital Association network, all but 1 institution offered pharmacogenetic testing for at least 1 clinical scenario, with 7 of 13 institutions (54%) using pharmacogenetic testing “regularly.”^3^ Tertiary pediatric institutions that use pharmacogenetic testing routinely have published on their experiences, which vary from automated testing of specific gene-drug pairs for all pediatric inpatients to specific units or specific testing of patients with specific diagnoses.^4,5,6,7,8,9,10,11,12,13,14,15^

Despite the increasing evidence of the association of pharmacogenetic testing with benefits among pediatric patients, common implementation barriers include cost, local testing availability, and testing platform selection.^3^ The decision to invest in pharmacogenetic testing implementation could be guided by better data on which drugs with the highest level of evidence for pharmacogenetic-guided dosing are most frequently prescribed or dispensed, the most frequently implicated genes that are known to influence medication dosing, and in which subpopulations these drugs are prescribed.

In 2019, when there were only 38 CPIC level A medications, Ramsey et al^16^ found that the annual prescribing prevalence of at least 1 CPIC level A drug ranged from approximately 8 to 11 prescriptions per 100 pediatric patients across 16 health systems from 2011 to 2017, with ondansetron the most commonly prescribed drug. We sought to investigate the prevalence of outpatient dispensing of CPIC level A medications among Medicaid-insured pediatric patients and examine trends of these dispensings over time. In addition, we determined the genes that were most frequently implicated as being associated with the dosing of these medications and described the characteristics of youths who were dispensed at least 1 CPIC level A medication.

## Methods

### Data Source, Study Design, and Study Population

We conducted serial cross-sectional studies using the Marketscan Medicaid database (Merative) from 2011 to 2019 and report our study following the Strengthening the Reporting of Observational Studies in Epidemiology (STROBE) reporting guideline. This deidentified dataset contains patient information from 10 to 12 states (variable by year) and includes claims from only outpatient and retail or mail order pharmacy fills (ie, dispensings). We limited analysis to patients aged 0 to 18 years with 11 or more months of continuous enrollment within a calendar year. This study was not considered human participants research by the policy and procedures of the Cincinnati Children’s Hospital Medical Center Institutional Review Board and so was exempted from requirements for review and informed consent.

### Definitions of Medications With High Level of Evidence for Pharmacogenetic-Guided Dosing and Associated Genes

We defined medications with a high level of evidence for pharmacogenetic-guided dosing (PGx drugs) as those that had CPIC level A evidence as of August 2022.^2^ CPIC is an international consortium that systemically grades the evidence for prescribing recommendations based on pharmacogenetic testing. For a level A assignment, CPIC members perform a comprehensive literature review and determine that there is sufficient evidence for at least 1 prescribing action to be recommended. A total of 67 medications were identified on the date that the CPIC website was queried (August 1, 2022). We also included sertraline, a commonly prescribed selective serotonin reuptake inhibitor (SSRI), given that an updated guideline was going to be published in 2023 designating sertraline as having CPIC level A evidence.^17^ A total of 68 medications were queried in the Medicaid database. These medications, as well as associated genes with level A evidence, are shown in eTable 1 in Supplement 1.

### Statistical Analysis

#### Prevalence of PGx Drug Dispensing

The number of unique youths dispensed at least 1 PGx drug and the number and percentage of unique youths dispensed each PGx drug were determined. This was found for each year between 2011 and 2019.

#### Rate of Dispensing of PGx Drugs by Gene

PGx drugs were grouped by their associated genes for which there was CPIC level A evidence to guide dosing on the basis of genetic variance. Given that 1 patient may be dispensed multiple medications affected by the same gene, a rate of dispensing of PGx drugs per 100 000 youths was determined for each gene for the year 2019. This analysis was limited to the most recent year to provide the most relevant information for implementing pharmacogenetic testing.

#### Demographics and Clinical Characteristics of Youths Dispensed PGx Drugs

Demographic data (ie, sex and race and ethnicity) and clinical characteristics (ie, number of noncomplex chronic conditions [non-CCCs], number of youths with CCCs, and number of mental health conditions) were extracted for the entire population queried, for youths who were not dispensed any PGx drugs, and for youths dispensed at least 1 PGx drug in 2019. This analysis was limited to 2019 because individual patient–level information is not available to track a single patient over multiple years. Race and ethnicity data in the database are collected according to each state’s Medicaid policies and may vary across states. Race and ethnicity are combined as a single variable in the database in response to an optional question at Medicaid enrollment. Race and ethnicity are categorized in the database as Hispanic or Latino, non-Hispanic Black, non-Hispanic White, and other (details of races and ethnicities included in this category were not available). Race and ethnicity are reported in this study to demonstrate that our findings were among a cohort with similar makeup as the US pediatric population for generalizability purposes. Non-CCCs were identified using the Chronic Condition Indicator from the Agency of Healthcare Quality; these were defined as conditions expected to last at least 12 months that required ongoing intervention with medical products, services, or equipment or caused limitations on self-care, independent living, or social interactions.^18^ Owing to specific interest in implementing pharmacogenetic testing in patients with medical complexity, we determined the percentage of patients with CCCs, which were defined using the pediatric CCCs classification system version 2.^19^ Mental health conditions, including but not limited to diagnoses of depressive and anxiety disorders, intellectual disability, and neurocognition disorders, were identified through *International Statistical Classification of Diseases, Tenth Revision, Clinical Modification *(*ICD-10-CM*) codes.^20^ Frequencies and percentages were compared using χ^2^ tests with SAS statistical software version 9.4 (SAS Institute). *P* values were 2-sided, and *P* < .05 was considered significant. Data were analyzed from August through December 2022.

## Results

### Prevalence of PGx Drug Dispensing

The number of Medicaid-insured youths queried ranged by year from 2 078 683 in 2011 to 4 641 494 youths in 2017 (Figure, A), including 4 126 349 youths (median [IQR] age, 9 [5-13] years; 2 129 926 males [51.6%]; 1 282 483 Black [31.1%], 507 693 Hispanic [12.3%], and 1 767 366 White [42.8%]) in the 2019 cohort (Table 1). In 2019, nearly 50% of youths (1 891 989 youths [45.8%]) had at least 1 chronic condition, with 724 472 youths (17.6%) identified as those with CCCs. The proportion of youths dispensed at least 1 PGx drug increased 28.8%, from 289 709 youths (13.9%; 95% CI, 13.8%-14.0%) in 2011 to 740 072 youths (17.9%; 95% CI, 17.9%-18.0%) in 2019 (Figure, A). Of 68 total drugs, 53 drugs (77.9%) were dispensed to less than 0.1% of youths (eTable 2 in Supplement 1).

**Figure.**
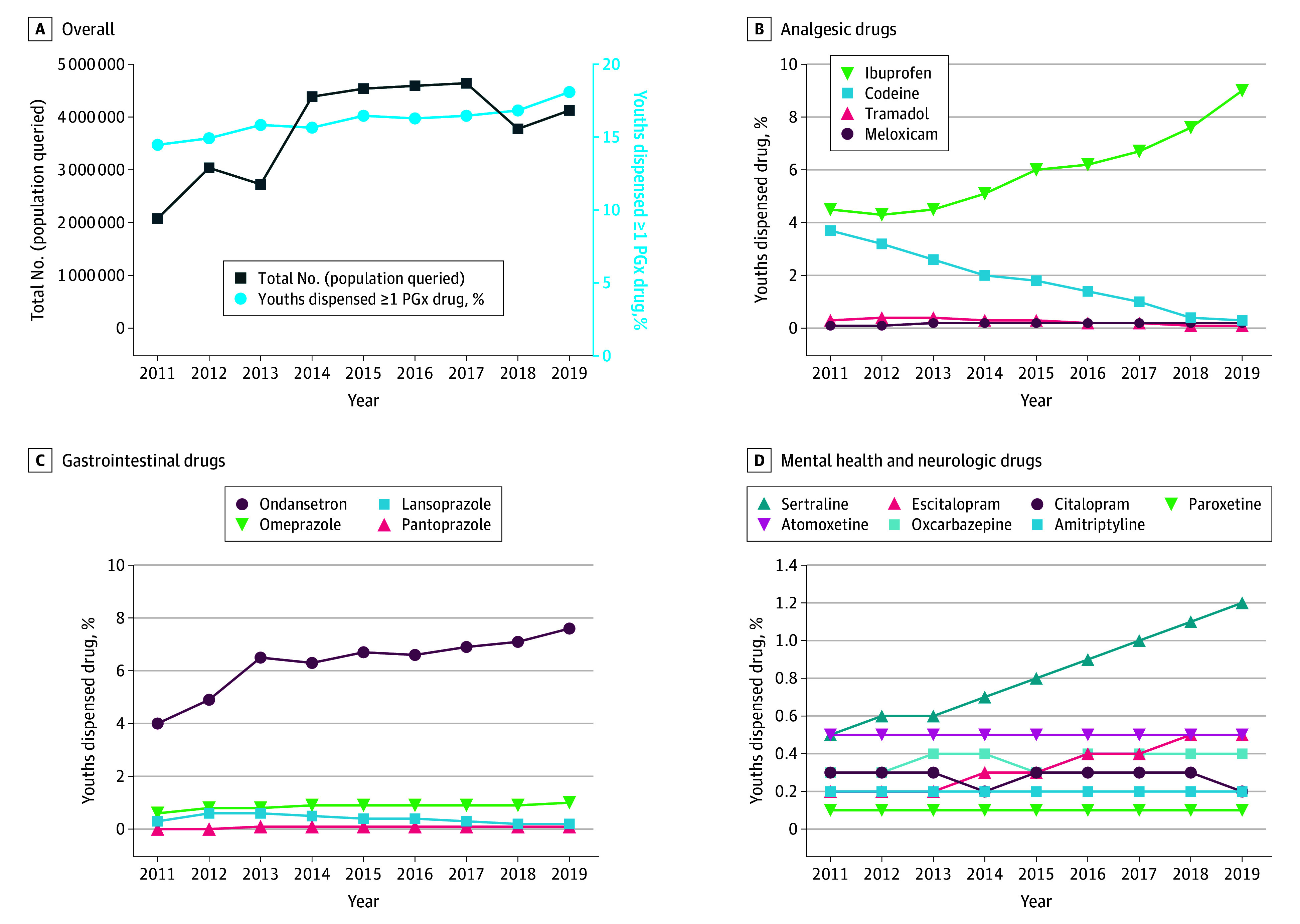
Youths Dispensed Drugs With High Level of Pharmacogenetic Evidence Proportions of youths in the Truven Medicaid database who were dispensed 1 or more medications with a high level of evidence for pharmacogenetic-guided dosing (PGx drugs) from 2011 to 2019 are given (A) overall and grouped by (B) analgesia, (C) gastrointestinal, and (D) mental health and neurologic indications. In A, the dark blue line with squares is associated with the left y-axis and the light blue line with circles is associated with the right y-axis. Only drugs for which at least 0.1% of the population was dispensed the drug in 2019 are shown.

**Table 1.** Patient Demographics and Clinical Characteristics in 2019

Characteristic	Patients in 2019, No. (%)		
	All patients aged <18 y (N = 4 126 349)	Dispensed 0 PGx drugs (n = 3 386 277 [82.1%])^a^	Dispensed ≥1 PGx drug (n = 740 072 [17.9%])^a^
Age, y			
Median (IQR)	9 (5-13)	9 (5-13)	9 (4-14)
0-1	256 548 (6.2)	203 675 (6.0)	52 873 (7.1)
2-4	727 723 (17.6)	591 610 (17.5)	136 113 (18.4)
5-11	1 744 711 (42.3)	1 470 859 (43.4)	273 852 (37)
12-17	1 397 367 (33.9)	1 120 133 (33.1)	277 234 (37.5)
Sex			
Male	2 129 926 (51.6)	1 763 524 (52.1)	366 402 (49.5)
Female	1 996 423 (48.4)	1 622 753 (47.9)	373 670 (50.5)
Race			
Black	1 282 483 (31.1)	1 073 160 (31.7)	209 323 (28.3)
Hispanic	507 693 (12.3)	436 674 (12.9)	71 019 (9.6)
White	1 767 366 (42.8)	1 416 516 (41.8)	350 850 (47.4)
Other^b^	269 877 (6.5)	224 167 (6.6)	45 710 (6.2)
Missing	298 930 (7.2)	235 760 (7.0)	63 170 (8.5)
No. of chronic conditions			
0	2 234 360 (54.1)	2 004 733 (59.2)	229 627 (31)
1	1 167 578 (28.3)	918 393 (27.1)	249 185 (33.7)
2-3	631 980 (15.3)	420 200 (12.4)	211 780 (28.6)
≥4	92 431 (2.2)	42 951 (1.3)	49 480 (6.7)
Complex chronic condition			
No	3 401 877 (82.4)	2 739 691 (80.9)	662 186 (89.5)
Yes	724 472 (17.6)	646 586 (19.1)	77 886 (10.5)
Number of mental health conditions			
0	3 152 514 (76.4)	2 694 064 (79.6)	458 450 (61.9)
1	506 870 (12.3)	391 397 (11.6)	115 473 (15.6)
2-3	366 243 (8.9)	249 906 (7.4)	116 337 (15.7)
≥4	100 722 (2.4)	50 910 (1.5)	49 812 (6.7)
No. of PGx drugs dispensed			
0	3 386 277 (82.1)	3 386 277 (100)	0
1	606 190 (14.7)	0	606 190 (81.9)
2-3	130 053 (3.2)	0	130 053 (17.6)
≥4	3829 (0.1)	0	3829 (0.5)

Abbreviation: PGx drug, medication with a high level of evidence of pharmacogenetic-guided dosing.

^a^ *P* values comparing patients dispensed 0 PGx drugs with those dispensed 1 or more PGx drug using χ^2^ tests were all <.001.

^b^ Other race and ethnicity was defined based on each state’s Medicaid policies, but this represents individuals who were not Black, Hispanic, or White.

Medications were grouped by indications, including those used for analgesia (Figure, B), gastrointestinal conditions (Figure, C), and mental health or neurologic conditions (Figure, D). For drugs used for analgesia, codeine dispensing decreased from 77 805 youths (3.7%; 95% CI, 3.7%-3.8%) in 2011 to 10 965 youths (0.3%; 95% CI, 0.2%-0.3%) in 2019. Ibuprofen dispensing concurrently increased from 94 436 youths (4.5%; 95% CI, 4.5%-4.6%) in 2011 to 371 749 youths (9.0%; 95% CI, 8.9%-9.1%) in 2019. For drugs with gastrointestinal disorder indications, ondansetron dispensing nearly doubled, from 83 292 youths (4.0%; 95% CI, 3.9%-4.1%) in 2011 to 314 960 youths (7.6%; 95% CI, 7.6%-7.7%) in 2019, and there was a consistent increase in the dispensing of the proton pump inhibitor (PPI) omeprazole. Lansoprazole, another PPI, had an initial increase in dispensing from 2011 to 2012 but then decreased over time. Among mental health drugs, sertraline and escitalopram, SSRIs used for depression, anxiety, or both in youths, showed a steady increase in dispensing over time.

### Rate of Dispensing of PGx Drugs by Gene

Grouping drugs by the genes for which there was level A evidence for pharmacogenetic-guided dosing, we found that *CYP2C9* was the most frequently implicated gene, with a rate of 9197.0 dispensings (95% CI, 9167.7-9226.3 dispensings) of associated PGx drugs per 100 000 youths in 2019 (Table 2). *CYP2D6* had a rate of 8731.5 dispensings (95% CI, 8702.5-8759.5 dispensings) of associated PGx drugs per 100 000 youths, and *CYP2C19* had a rate of 3426.8 dispensings (95% CI, 3408.1-3443.9 dispensings) per 100 000 youths. Remaining implicated genes had a dispensing rate of fewer than 500 dispensings per 100 000 youths.

**Table 2.** Most Frequently Implicated Genes

Gene^a^	Associated PGx drugs	Dispensing rate, No./100 000 youths^b^
*CYP2C9 *	Celecoxib, flurbiprofen, fluvastatin, fosphenytoin, ibuprofen, lornoxicam, meloxicam, phenytoin, piroxicam, siponimod, tenoxicam, and warfarin	9197.0
*CYP2D6 *	Amitriptyline, atomoxetine, codeine, nortriptyline, ondansetron, paroxetine, pitolisant, tamoxifen, tramadol, and tropisetron	8731.5
*CYP2C19 *	Amitriptyline, citalopram, clopidogrel, escitalopram, lansoprazole, omeprazole, pantoprazole, sertraline, and voriconazole	3426.8
*HLA-B *	Abacavir, allopurinol, carbamazepine, fosphenytoin, oxcarbazepine, and phenytoin	453.2
*CACNA1S *	Desflurane, enflurane, halothane, isoflurane, methoxyflurane, sevoflurane, and succinylcholine	39.5
*RYR1 *	Desflurane, enflurane, halothane, isoflurane, methoxyflurane, sevoflurane, and succinylcholine	39.5
*NUDT15 *	Azathioprine, mercaptopurine, and thioguanine	33.4
*TPMT *	Azathioprine, mercaptopurine, and thioguanine	33.4
*CYP3A5 *	Tacrolimus	30.8
*HLA-A *	Carbamazepine	29.9
*MT-RNR1 *	Amikacin, gentamicin, kanamycin, paromomycin, plazomicin, streptomycin, and tobramycin	
*SLCO1B1 *	Atorvastatin, fluvastatin, lovastatin, pitavastatin, pravastatin, rosuvastatin, and simvastatin	29.5
*CFTR *	Ivacaftor	13.6
*CYP4F2 *	Warfarin	8.1
*VKORC1 *	Warfarin	8.1
*ABCG2 *	Rosuvastatin	1.7

Abbreviation: PGx drug, medication with a high level of evidence of pharmacogenetic-guided dosing.

^a^ Only genes with rates higher than 1 dispensing per 100 000 youths are shown. Genes included in analysis but not shown because the rate of prescription was less than 1 dispensing in 100 000 youths included *CYP2B6*, *DPYD*, *G6PD*, *IFNL3*, *IFNL4*, and *UGT1A1.*

^b^ Most frequently implicated genes are shown based on the dispensing rate of PGx drugs per 100 000 youths in 2019.

### Demographics and Clinical Characteristics of Youths Dispensed PGx Drugs

We compared demographics and clinical characteristics of 740 072 youths dispensed at least 1 PGx drug with those of 3 386 277 youths who were not dispensed any PGx drug in 2019 (Table 1). While the median age of the groups was similar, there was a higher percentage of adolescents (ages 12-17 years) in the group dispensed at least 1 PGx drug (277 234 youths [37.5%; 95% CI, 37.3%-37.6%]) compared with the group not dispensed any PGx drug (1 120 133 youths [33.1%; 95% CI, 33.0%-33.1%]; *P* < .001). There was also a higher percentage of females in the group dispensed at least 1 PGx drug (373 670 females [50.5%; 95% CI, 50.3%-50.6%] vs 1 622 753 females [47.9%; 95% CI, 47.8%-48.0%]; *P* < .001). There was a higher percentage of youths with at least 1 chronic medical condition (510 445 youths [69.0%; 95% CI, 68.8%-69.1%] vs 1 381 544 youths [40.8%; 95% CI, 40.7%-40.9%]; *P* < .001) or mental health condition (281 622 youths [38.1%; 95% CI, 37.9%-38.2%] vs 692 213 youths [20.4%; 95% CI, 20.3%-20.5%]; *P* < .001) in the cohort dispensed at least 1 PGx drug. There was a lower percentage of youths with 1 or more CCCs in the cohort dispensed at least 1 PGx drug (77 886 youths [10.5%; 95% CI, 10.4%-10.6%] vs 646 586 youths [19.1%; 95% CI, 19.0%-19.1%]; *P* < .001). Among all youths with CCCs, 10.8% were dispensed at least 1 PGx drug, while among 3 401 877 youths without CCCs, 662 186 youths (19.5%) were dispensed at least 1 PGx drug.

## Discussion

This cross-sectional study found that dispensing of PGx drugs per CPIC guidelines increased among Medicaid-enrolled youths. As expected, our prevalence of dispensing was higher than that reported in the 2019 study by Ramsey et al^16^ during comparable years given that we included 30 more medications that have since been added as having a high level of evidence for pharmacogenetic-guided dosing between 2019 and 2022. Genes that were associated with frequently dispensed medications included *CYP2C9*, *CYP2D6*, and *CYP2C19*. These findings may guide institutional decisions on prioritizing drug-gene pairs for pharmacogenetic testing.

Ibuprofen was the most highly dispensed PGx drug in 2018 and 2019. It is likely we underestimated use of ibuprofen given that it is often obtained over the counter. However, it was interesting that dispensing of ibuprofen increased concurrently as codeine use decreased over the decade. The decrease in codeine use coincided with warnings from the Food and Drug Administration (FDA) about use of codeine in youths for pain or cough in 2013. This warning was strengthened to a contraindication of use in children younger than age 12 years in 2017^21^ due to increased adverse events, including death in patients who were *CYP2D6* ultrarapid metabolizers and among children with abnormal respiratory status. Similarly, we also found decreasing tramadol dispensings coinciding with changes to FDA recommendations contraindicating its use to treat pain in children younger than 12 years. The increase in ibuprofen dispensings may have been a response to severe restrictions on codeine and tramadol prescriptions in order to avoid any opioid prescription. Additional studies to examine trends of prescriptions of opioids without a high level of evidence for pharmacogenetic-guided dosing (ie, not codeine or tramadol) would be of interest.

In addition to finding a high number of dispensings for analgesics, we found that drugs for mental health disorders were dispensed for a significant proportion of youths. We found a higher prevalence of youths with mental health conditions among those dispensed at least 1 PGx drug and an increasing use of SSRIs, such as sertraline and escitalopram, over time, suggesting that institutions could focus implementation of PGx testing in outpatient psychiatry clinics or inpatient psychiatric units. At 1 institution, all patients admitted to the inpatient psychiatry unit have had routine PGx testing since 2005.^4^ Institutions could also choose to test genes that are associated with the most highly prescribed medications. In our findings, these included *CYP2C9*, *CYP2D6*, and *CYP2C19*. Investment in these 3 genes may be associated with changes in prescriptions of more than 10 drugs that are prescribed frequently to youths. Alternatively, institutions could focus on genes involved in the metabolism of drugs with narrow therapeutic windows. Low concentrations of these drugs can lead to rapid disease progression, and high concentrations may result in severe toxic effects, even if prevalence of prescriptions are not very high. Examples of these drugs include thiopurines (eg, azathioprine, mercaptopurine, and thioguanine, which are associated with *TPMT* and *NUDT15*), tacrolimus (associated with *CYP3A5)*, and voriconazole (associated with *CYP2C19).* These drug-gene pairs are indeed the most frequently tested in pediatric institutions that have implemented PGx testing.^3^

An increasing number of youths with medical complexity are living longer, and they are often subjected to polypharmacy, so there may be increasing benefits associated with PGx testing in this population. In 1 institution, more than two-thirds of more than 800 patients in a complex care program were prescribed at least 1 medication with an established PGx association.^22^ Within a subset of these 50 patients for whom the institution specifically performed genetic testing, the institution identified that half of these patient had variants in pharmacogenes associated with dosing for commonly prescribed medications in patients with medical complexity. Based on these findings, we expected that a large proportion of patients with CCCs in our study would be dispensed PGx drugs. However, we found that 77 886 of 724 472 youths (10.8%) identified as having CCCs were dispensed at least 1 PGx drug. Among 740 072 youths dispensed at least 1 PGx drug, 10.5% had CCCs, compared with 19.1% of 3 386 277 youths dispensed no PGx drugs. One major reason for these unexpected findings may be that the population that qualifies for a specific institution’s complex care program may be different than the population identified as having CCCs in the Medicaid database. That study^22^ also included CPIC level A and level B medications, which may account for the higher prevalence. We found that a smaller percentage of patients with CCCs were dispensed PGx drugs compared with those without CCC in our study (10.8% vs 662 186 patients [19.5%]); 1 reason for this finding is that patients with CCCs may be less likely to be diagnosed with mental health conditions, such as depression and anxiety, for which SSRIs are prescribed, while mental or behavioral health disorders are common among youths with CCCs.^23^

### Limitations

There are limitations to this study. The dataset contains only youths who were insured by Medicaid, and so findings may not be generalizable to youths with private insurance. However, this population represents an ideal group of patients in which to implement pharmacogenetic testing given that Medicaid covers pharmacogenetic tests for approved medications that are medically necessary and carry CPIC level A or B evidence. This reduces cost as a potential barrier to implementation.^24^ In addition, many youths with high medical complexity are insured by Medicaid.

With our data, we were unable to track patients from year to year to determine how often pharmacogenetic test results could be reused with different prescriptions that are associated with the same gene. Several of the medications, including ibuprofen and PPIs, can be obtained over the counter, which likely led to underestimation of their use. These dispensings include as needed and chronic medications given that prescriptions for both types of medications could benefit from pharmacogenetic testing. It is likely that ondansetron and ibuprofen, 2 prescriptions associated with the increase in the prevalence of PGx drug dispensing over time, were prescribed more frequently as needed than chronically. However, for nonsteroidal anti-inflammatory drugs like ibuprofen, there is evidence that risks of upper gastrointestinal bleeding are similar among new and chronic users.^25^ Therefore, pharmacogenetic testing could mitigate these risks for as needed and chronic use. Additionally, without knowledge of each youth’s genotype, it is unknown how many medication prescriptions would have been affected if PGx testing had been performed, although studies have indicated that nearly every person carries at least 1 PGx gene with an actionable variant.^26^

## Conclusions

In this cross-sectional study of Medicaid-insured youths, there was a nearly 30% increase in the proportion of youths dispensed medications with a high level of evidence for pharmacogenetic-guided dosing over the last 10 years. This finding suggests that institutions interested in implementing PGx testing could consider focusing on medications with an increasing number of prescriptions (eg, mental health drugs) or genes that are highly implicated (ie, cytochrome P450 genes) for testing during hospitalization.

## Notes

Supplement 1. **eTable 1.** Clinical Pharmacogenetics Implementation Consortium Level A Drugs Queried in August 2022 and Associated Genes **eTable 2.** Prevalence of Dispensings for Drugs With High Level of Pharmacogenetic Evidence Among Youths with Medicaid 2011-2019

Supplement 2.
Data Sharing Statement
