# Supplementary material for: Opportunities for Pharmacogenetic Testing to Guide Dosing of Medications in Youths With Medicaid
Source: JAMA Netw Open. 2024 Feb 13;7(2):e2355707. doi: 10.1001/jamanetworkopen.2023.55707 (PMC10865156; doi:10.1001/jamanetworkopen.2023.55707)
Supplement: Supplement 1. — eTable 1. Clinical Pharmacogenetics Implementation Consortium Level A Drugs Queried in August 2022 and Associated Genes eTable 2. Prevalence of Dispensings for Drugs With High Level of Pharmacogenetic Evidence Among Youths with Medicaid 2011-2019 [file jamanetwopen-e2355707-s001.pdf]

Supplemental Online Content

Tang Girdwood S, Hall M, Antoon JW, et al. Opportunities for pharmacogenetic testing to guide dosing of medications in youths with Medicaid. *JAMA Netw Open*. 2024;7(2):e2355707. doi:10.1001/jamanetworkopen.2023.55707

eTable 1. Clinical Pharmacogenetics Implementation Consortium Level A Drugs Queried in August 2022 and Associated Genes

eTable 2. Prevalence of Dispensings for Drugs With High Level of Pharmacogenetic Evidence Among Youths with Medicaid 2011-2019

This supplemental material has been provided by the authors to give readers additional information about their work.

© 2024 Tang Girdwood S et al. *JAMA Network Open.*

Supplementary Table 1: List of CPIC Level A Drugs Queried in August 2022 and Their Associated Genes

CPIC Level A Drug	Associated Level A Gene(s)
abacavir	HLA-B
allopurinol	HLA-B
amikacin	MT-RNR1
amitriptyline	CYP2C19, CYP2D6
atazanavir	UGT1A1
atomoxetine	CYP2D6
atorvastatin	SLCO1B1
azathioprine	NUDT15, TPMT
capecitabine	DPYD
carbamazepine	HLA-A, HLA-B
celecoxib	CYP2C9
citalopram	CYP2C19
clopidogrel	CYP2C19
codeine	CYP2D6
desflurane	CACNA1S, RYR1
efavirenz	CYP2B6
enflurane	CACNA1S, RYR1
escitalopram	CYP2C19
fluorouracil	DPYD
flurbiprofen	CYP2C9
fluvastatin	CYP2C9, SLCO1B1
fosphenytoin	CYP2C9, HLA-B
gentamicin	MT-RNR1
halothane	CACNA1S, RYR1
ibuprofen	CYP2C9
irinotecan	UGT1A1
isoflurane	CACNA1S, RYR1
ivacaftor	CFTR
kanamycin	MT-RNR1
lansoprazole	CYP2C19
lornoxicam	CYP2C9
lovastatin	SLCO1B1
meloxicam	CYP2C9
mercaptopurine	NUDT15, TPMT
methoxyflurane	CACNA1S, RYR1

© 2024 Tang Girdwood S et al. *JAMA Network Open.*

nortriptyline	CYP2D6
omeprazole	CYP2C19
ondansetron	CYP2D6
oxcarbazepine	HLA-B
pantoprazole	CYP2C19
paromomycin	MT-RNR1
paroxetine	CYP2D6
peginterferon_alfa_2a	IFNL3, IFNL4
peginterferon_alfa_2b	IFNL3, IFNL4
phenytoin	CYP2C9, HLA-B
piroxicam	CYP2C9
pitavastatin	SLCO1B1
pitolisant	CYP2D6
plazomicin	MT-RNR1
pravastatin	SLCO1B1
rasburicase	G6PD
rosuvastatin	ABCG2, SLCO1B1
sertraline	CYP2C19
sevoflurane	CACNA1S, RYR1
simvastatin	SLCO1B1
siponimod	CYP2C9
streptomycin	MT-RNR1
succinylcholine	CACNA1S, RYR1
tacrolimus	CYP3A5
tafenoquine	G6PD
tamoxifen	CYP2D6
tenoxicam	CYP2C9
thioguanine	NUDT15, TPMT
tobramycin	MT-RNR1
tramadol	CYP2D6
tropisetron	CYP2D6
voriconazole	CYP2C19
warfarin	CYP2C9, CYP4F2, VKORC1

© 2024 Tang Girdwood S et al. *JAMA Network Open.*

Supplementary Table 2: Prevalence of Dispensings for PGx Drugs Among Children with Medicaid from 2011-2019

	2011	2012	2013	2014	2015	2016	2017	2018	2019
**N (entire population queried)**	2,078,683	3,038,981	2,727,587	4,387,875	4,538,116	4,591,241	4,641,494	3,778,285	4,126,349
**N (number of patients dispensed at least 1 PGx drug)**	289,709	436,886	421,211	665,286	728,417	729,543	747,192	624,235	740,072
**% of population queried dispensed at least 1 PGx drug**	13.9%	14.4%	15.4%	15.2%	16.1%	15.9%	16.1%	16.5%	17.9%
**Number of patients dispensed the following (% of population queried)**									
**abacavir**	83 (0)	106 (0)	99 (0)	117 (0)	132 (0)	131 (0)	125 (0)	93 (0)	90 (0)
**allopurinol**	46 (0)	63 (0)	53 (0)	102 (0)	113 (0)	137 (0)	157 (0)	158 (0)	150 (0)
**amikacin**	5 (0)	20 (0)	18 (0)	35 (0)	41 (0)	45 (0)	46 (0)	47 (0)	50 (0)
**amitriptyline**	4581 (0.2)	6532 (0.2)	6387 (0.2)	10019 (0.2)	11010 (0.2)	11368 (0.2)	11293 (0.2)	8674 (0.2)	8918 (0.2)
**atazanavir**	45 (0)	62 (0)	56 (0)	68 (0)	56 (0)	46 (0)	29 (0)	12 (0)	12 (0)
**atomoxetine**	10765 (0.5)	15671 (0.5)	14007 (0.5)	21329 (0.5)	21706 (0.5)	21591 (0.5)	21419 (0.5)	17439 (0.5)	19088 (0.5)
**atorvastatin**	141 (0)	172 (0)	238 (0)	458 (0)	568 (0)	676 (0)	736 (0)	635 (0)	687 (0)
**azathioprine**	105 (0)	132 (0)	140 (0)	285 (0)	528 (0)	538 (0)	495 (0)	385 (0)	457 (0)
**capecitabine**	0 (0)	2 (0)	0 (0)	1 (0)	0 (0)	0 (0)	0 (0)	1 (0)	1 (0)
**carbamazepine**	2717 (0.1)	3347 (0.1)	2627 (0.1)	2980 (0.1)	2668 (0.1)	2257 (0)	1858 (0)	1388 (0)	1234 (0)
**celecoxib**	157 (0)	241 (0)	197 (0)	336 (0)	283 (0)	409 (0)	456 (0)	376 (0)	455 (0)
**citalopram**	6930 (0.3)	8909 (0.3)	7144 (0.3)	10485 (0.2)	12436 (0.3)	12560 (0.3)	12233 (0.3)	9641 (0.3)	9514 (0.2)
**clopidogrel**	21 (0)	44 (0)	46 (0)	69 (0)	71 (0)	73 (0)	87 (0)	80 (0)	119 (0)
**codeine**	77805 (3.7)	98381 (3.2)	70836 (2.6)	87858 (2)	80092 (1.8)	65118 (1.4)	44799 (1)	16312 (0.4)	10965 (0.3)
**desflurane**	0 (0)	0 (0)	0 (0)	0 (0)	6 (0)	49 (0)	19 (0)	10 (0)	51 (0)
**efavirenz**	61 (0)	54 (0)	48 (0)	54 (0)	43 (0)	28 (0)	18 (0)	10 (0)	9 (0)
**enflurane**	0 (0)	0 (0)	0 (0)	0 (0)	0 (0)	0 (0)	0 (0)	0 (0)	0 (0)
**escitalopram**	4117 (0.2)	6488 (0.2)	6603 (0.2)	12304 (0.3)	14466 (0.3)	16704 (0.4)	19198 (0.4)	18010 (0.5)	22309 (0.5)
**fluorouracil**	0 (0)	2 (0)	1 (0)	0 (0)	0 (0)	1 (0)	1 (0)	0 (0)	0 (0)
**flurbiprofen**	57 (0)	81 (0)	54 (0)	92 (0)	55 (0)	41 (0)	33 (0)	20 (0)	8 (0)
**fluvastatin**	0 (0)	0 (0)	0 (0)	0 (0)	1 (0)	2 (0)	0 (0)	0 (0)	0 (0)
**fosphenytoin**	0 (0)	0 (0)	10 (0)	13 (0)	17 (0)	15 (0)	16 (0)	15 (0)	23 (0)
**gentamicin**	155 (0)	196 (0)	189 (0)	301 (0)	273 (0)	280 (0)	249 (0)	183 (0)	199 (0)

© 2024 Tang Girdwood S et al. *JAMA Network Open.*

**halothane**	0 (0)	0 (0)	0 (0)	0 (0)	0 (0)	0 (0)	0 (0)	0 (0)	0 (0)
**ibuprofen**	94436 (4.5)	130821 (4.3)	122779 (4.5)	225254 (5.1)	271531 (6)	284622 (6.2)	312753 (6.7)	288602 (7.6)	371749 (9)
**irinotecan**	5 (0)	11 (0)	8 (0)	13 (0)	17 (0)	15 (0)	17 (0)	14 (0)	18 (0)
**isoflurane**	0 (0)	0 (0)	0 (0)	0 (0)	0 (0)	0 (0)	0 (0)	0 (0)	0 (0)
**ivacaftor**	0 (0)	29 (0)	26 (0)	47 (0)	146 (0)	214 (0)	338 (0)	366 (0)	559 (0)
**kanamycin**	0 (0)	0 (0)	0 (0)	0 (0)	0 (0)	0 (0)	0 (0)	0 (0)	0 (0)
**lansoprazole**	7106 (0.3)	17897 (0.6)	16080 (0.6)	23005 (0.5)	19450 (0.4)	16905 (0.4)	13033 (0.3)	8267 (0.2)	8152 (0.2)
**lornoxicam**	0 (0)	0 (0)	0 (0)	0 (0)	0 (0)	0 (0)	0 (0)	0 (0)	0 (0)
**lovastatin**	82 (0)	80 (0)	58 (0)	61 (0)	57 (0)	38 (0)	39 (0)	34 (0)	21 (0)
**meloxicam**	2354 (0.1)	3897 (0.1)	4589 (0.2)	7452 (0.2)	7863 (0.2)	7854 (0.2)	8155 (0.2)	6418 (0.2)	6727 (0.2)
**mercaptopurine**	472 (0)	673 (0)	652 (0)	874 (0)	947 (0)	949 (0)	926 (0)	786 (0)	782 (0)
**methoxyflurane**	0 (0)	0 (0)	0 (0)	0 (0)	0 (0)	0 (0)	0 (0)	0 (0)	0 (0)
**nortriptyline**	448 (0)	700 (0)	643 (0)	902 (0)	987 (0)	1138 (0)	1364 (0)	1141 (0)	1019 (0)
**omeprazole**	11725 (0.6)	24540 (0.8)	22929 (0.8)	37890 (0.9)	42550 (0.9)	42765 (0.9)	42802 (0.9)	35417 (0.9)	39261 (1)
**ondansetron**	83292 (4)	150206 (4.9)	176713 (6.5)	275327 (6.3)	304669 (6.7)	303788 (6.6)	319631 (6.9)	266948 (7.1)	314960 (7.6)
**oxcarbazepine**	6995 (0.3)	8873 (0.3)	10109 (0.4)	15877 (0.4)	15660 (0.3)	16993 (0.4)	17770 (0.4)	15513 (0.4)	17010 (0.4)
**pantoprazole**	359 (0)	1212 (0)	1411 (0.1)	2787 (0.1)	3615 (0.1)	4443 (0.1)	3803 (0.1)	3225 (0.1)	4115 (0.1)
**paromomycin**	9 (0)	8 (0)	7 (0)	11 (0)	7 (0)	4 (0)	19 (0)	3 (0)	19 (0)
**paroxetine**	1176 (0.1)	1849 (0.1)	1829 (0.1)	2719 (0.1)	2747 (0.1)	2638 (0.1)	2613 (0.1)	2256 (0.1)	2386 (0.1)
**peginterferon_alfa_2a**	0 (0)	4 (0)	5 (0)	1 (0)	0 (0)	1 (0)	1 (0)	0 (0)	0 (0)
**peginterferon_alfa_2b**	0 (0)	0 (0)	0 (0)	0 (0)	2 (0)	5 (0)	5 (0)	3 (0)	5 (0)
**phenytoin**	411 (0)	455 (0)	363 (0)	466 (0)	391 (0)	344 (0)	302 (0)	195 (0)	192 (0)
**piroxicam**	156 (0)	169 (0)	121 (0)	122 (0)	104 (0)	67 (0)	51 (0)	13 (0)	10 (0)
**pitavastatin**	0 (0)	0 (0)	0 (0)	0 (0)	0 (0)	0 (0)	0 (0)	0 (0)	0 (0)
**pitolisant**	0 (0)	0 (0)	0 (0)	0 (0)	0 (0)	0 (0)	0 (0)	0 (0)	0 (0)
**plazomicin**	0 (0)	0 (0)	0 (0)	0 (0)	0 (0)	0 (0)	0 (0)	0 (0)	0 (0)
**pravastatin**	213 (0)	281 (0)	270 (0)	292 (0)	272 (0)	264 (0)	270 (0)	212 (0)	229 (0)
**rasburicase**	0 (0)	0 (0)	0 (0)	0 (0)	0 (0)	0 (0)	0 (0)	0 (0)	0 (0)
**rosuvastatin**	30 (0)	47 (0)	32 (0)	36 (0)	29 (0)	23 (0)	29 (0)	47 (0)	69 (0)
**sertraline**	10801 (0.5)	17341 (0.6)	17095 (0.6)	29610 (0.7)	37704 (0.8)	42536 (0.9)	46484 (1)	41470 (1.1)	48784 (1.2)
**sevoflurane**	0 (0)	4 (0)	3 (0)	69 (0)	139 (0)	1199 (0)	888 (0)	959 (0)	1161 (0)

© 2024 Tang Girdwood S et al. *JAMA Network Open.*

**simvastatin**	307 (0)	412 (0)	344 (0)	403 (0)	321 (0)	261 (0)	265 (0)	207 (0)	211 (0)
**siponimod**	0 (0)	0 (0)	0 (0)	0 (0)	0 (0)	0 (0)	0 (0)	0 (0)	0 (0)
**streptomycin**	0 (0)	0 (0)	0 (0)	0 (0)	0 (0)	0 (0)	2 (0)	4 (0)	2 (0)
**succinylcholine**	3 (0)	6 (0)	6 (0)	138 (0)	290 (0)	398 (0)	275 (0)	388 (0)	418 (0)
**tacrolimus**	533 (0)	795 (0)	773 (0)	1116 (0)	1132 (0)	1132 (0)	1148 (0)	1071 (0)	1271 (0)
**tafenoquine**	0 (0)	0 (0)	0 (0)	0 (0)	0 (0)	0 (0)	0 (0)	0 (0)	0 (0)
**tamoxifen**	10 (0)	15 (0)	8 (0)	22 (0)	20 (0)	25 (0)	37 (0)	31 (0)	36 (0)
**tenoxicam**	0 (0)	0 (0)	0 (0)	0 (0)	0 (0)	0 (0)	0 (0)	0 (0)	0 (0)
**thioguanine**	0 (0)	0 (0)	47 (0)	146 (0)	155 (0)	155 (0)	148 (0)	118 (0)	140 (0)
**tobramycin**	616 (0)	844 (0)	688 (0)	1104 (0)	1072 (0)	1001 (0)	945 (0)	823 (0)	962 (0)
**tramadol**	7136 (0.3)	10886 (0.4)	10112 (0.4)	13918 (0.3)	11742 (0.3)	10190 (0.2)	7801 (0.2)	4041 (0.1)	2910 (0.1)
**tropisetron**	0 (0)	0 (0)	0 (0)	0 (0)	0 (0)	0 (0)	0 (0)	0 (0)	0 (0)
**voriconazole**	98 (0)	115 (0)	106 (0)	127 (0)	112 (0)	123 (0)	131 (0)	126 (0)	187 (0)
**warfarin**	198 (0)	261 (0)	211 (0)	356 (0)	352 (0)	339 (0)	346 (0)	284 (0)	336 (0)

© 2024 Tang Girdwood S et al. *JAMA Network Open.*
