# Supplementary material for: Opportunities for Pharmacogenetic Testing to Guide Dosing of Medications in Youths With Medicaid
Source: JAMA Netw Open. 2024 Feb 13;7(2):e2355707. doi: 10.1001/jamanetworkopen.2023.55707 (PMC10865156; doi:10.1001/jamanetworkopen.2023.55707)
Supplement: Supplement 2. — Data Sharing Statement [file jamanetwopen-e2355707-s002.pdf]

Data Sharing Statement

Tang Girdwood. Opportunities for Pharmacogenetic Testing to Guide Dosing of Medications in Youths With Medicaid. *JAMA Netw Open*. Published online February 13, 2024. doi:10.1001/jamanetworkopen.2023.55707

Data
Data available: No
